# Hepatic KLF9 Deficiency Inhibits Dehydroepiandrosterone (DHEA)‐Induced Polycystic Ovary Syndrome via Liver‐Ovary Axis

**DOI:** 10.1002/advs.202508240

**Published:** 2025-09-23

**Authors:** Jianli Song, Zhaoqing Guan, Guiyi Ji, Jing Wang, Xiurui Yan, Yujie Zhang, Yinliang Zhang, Chunfang Ha, Rong Hu, Yongsheng Chang, Yaolin Zhang, Heng Fan

**Affiliations:** ^1^ Department of Reproductive Medicine General Hospital of Ningxia Medical University (The First Clinical Medical College of Ningxia Medical University) No. 804, Shengli Street Yinchuan Ningxia Hui Autonomous Region 750003 China; ^2^ Ningxia Key Laboratory of Stem Cell and Regenerative Medicine, Institute of Medical Sciences General Hospital of Ningxia Medical University No. 804, Shengli Street Yinchuan Ningxia Hui Autonomous Region 750003 China; ^3^ Department of Physiology and Pathophysiology, School of Basic Medical Sciences, Key Laboratory of Immune Microenvironment and Disease (Ministry of Education), Tianjin Key Laboratory of Cellular Homeostasis and Disease Tianjin Medical University No. 22, Qixiangtai Road Tianjin 300070 China; ^4^ Department of Gynecology General Hospital of Ningxia Medical University No. 804, Shengli Street Yinchuan Ningxia Hui Autonomous Region 750003 China; ^5^ Institute of Medical Sciences General Hospital of Ningxia Medical University No. 804, Shengli Street Yinchuan Ningxia Hui Autonomous Region 750003 China

**Keywords:** androgen, Klf9, liver‐ovary axis, PCOS

## Abstract

Hyperandrogenism is one of the key leading causes of polycystic ovary syndrome (PCOS), which is a complex metabolic disorder affecting 6% to 20% of women of reproductive age. However, the molecular pathogenetic mechanisms responsible for androgen excess in PCOS remain largely unknow. While most previous studies have specifically focused on ovarian tissue, few have evaluated the role of extraovarian organs in PCOS. Here, it is found that KLF9 expression is up‐regulated in murine primary hepatocytes treated with DHEA. Notably, the genetic ablation of *Klf9* in hepatocytes significantly alleviated the progression of DHEA induced PCOS in mouse. Conversely, hepatic *Klf9* transgenic mice displayed a spontaneous PCOS‐like phenotype. Mechanistically, hepatic KLF9 is directly activated by intranuclear AR and then directly binds to the promoter of *Srd5a1* and the gene loci of *Hsd3b3*, which encode the enzyme for the conversion of DHEA to dihydrotestosterone, to promote its transcription in the liver. Overall, our study indicated that the liver plays a vital role in the development of PCOS and that hepatic KLF9 might be a potential therapeutic target for PCOS.

## Graphical abstract

The study shows that liver is a previously unrecognized extraovarian organ that plays a key role about androgen homeostasis in the development of DHEA‐induced PCOS. DHEA is the most common precursor of active androgen, such as DHT, and KLF9 is required for the conversion of DHEA to DHT and hepatic KLF9 might be a potential therapeutic target for PCOS.

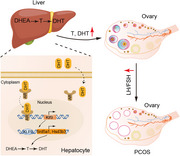

## 1 Introduction

PCOS is the most common metabolic endocrine disorder, is characterized by hyperandrogenism, ovulatory dysfunction and/or polycystic ovarian morphology (PCOM) and is the leading cause of subfertility in reproductive‐aged women.^[^ ^1^ ^]^ Recent data indicate that the incidence of PCOS is ≈6% to 20% worldwide, and is increasing as obesity increases.^[^ ^2^, ^3^, ^4^ ^]^ Patients diagnosed with PCOS are prone to type 2 diabetes, cardiovascular disease and psychological disorders,^[^ ^5^, ^6^, ^7^, ^8^ ^]^ all of which impose a heavy economic burden on afflicted individuals. Unfortunately, there is no effective treatment for PCOS except for controlling specific symptoms. Hyperandrogenemia is a salient feature of PCOS and is present in ≈80%–85% of patients diagnosed with PCOS.^[^ ^9^, ^10^ ^]^ According to the Rotterdam criteria, three of the four PCOS subtypes are characterized by hyperandrogenism.^[^ ^5^ ^]^ Compelling evidence indicates that androgen excess plays a prominent role in the development of PCOS.^[^ ^11^ ^]^ For example, the systemic administration of androgens could induce PCOS in animals including mice,^[^ ^12^ ^]^ rats,^[^ ^13^ ^]^ sheep,^[^ ^14^ ^]^ and monkeys.^[^ ^15^ ^]^ In addition, ≈25%–50% of PCOS patients with ovarian hyperandrogenism is accompanied by elevated dehydroepiandrosterone (DHEA) of adrenal origin.^[^ ^16^ ^]^ Thus, androgen excess can be considered a driver symptom of PCOS.

DHEA, dehydroepiandrosterone sulphate (DHEAS), androstenedione, and androstenediol, testosterone (T) and dihydrotestosterone (DHT) are different androgen forms. DHEA is the most common precursor of T and DHT. DHEA can be converted into androstenedione by 3β‐HSD, and then androstenedione can be converted into T by 17β‐HSD. Under the catalytic action of 5α‐reductase (SRD5A1/SRD5A2), T is then converted into DHT, which is ≈100‐fold more biologically active than T.^[^ ^17^ ^]^ In the cytoplasm, once DHT binds with the androgen receptor (AR), it can travel to the nuclear to regulate the target gene expression.^[^ ^18^ ^]^ However, whether the process of androgen conversion occurs in hepatocytes remains largely unclear.

The liver, a metabolic center enriched by enzymes that are responsible for a multitude of metabolic processes, is an important organ for maintaining metabolic and hormone homeostasis. In the liver, hepatocytes, which account for ≈60% of total liver cells, play a predominant role in maintaining liver functions. Growing evidence indicates that the liver can affect the initiation or progression of various diseases through interactions with multiple organs. For example, liver‐derived protein coagulation factor XI (FXI) protects against heart failure through the (BMP)–SMAD1/5 pathway,^[^ ^19^ ^]^ a finding that provides new evidence for a liver‐heart axis. In addition, hepatic PP2Acα exacerbates the progression of hepatic osteodystrophy disease, which highlights the importance of liver‐bone axis.^[^ ^20^ ^]^ Recent studies have suggested that organs other than the ovaries, such as the brain and adipose tissue, play a significant role in the development of PCOS.^[^ ^21^ ^]^ However, it remains unclear whether liver will affect the development of PCOS.

Krüppel‐like factor 9 (KLF9/Bteb1) belongs to the Krüppel‐like factor (KLF) family, which includes zinc‐finger‐containing transcription factors with highly conserved C‐terminal DNA‐binding domains.^[^ ^22^ ^]^ KLF9 is widely expressed in multiple tissues including liver and ovary. It has been reported that KLF9 is necessary for late‐phase neuronal maturation.^[^ ^23^ ^]^ Notably, emerging evidence indicates that KLF9 is a crucial transcription factor involved in glucose and lipid metabolism.^[^ ^24^, ^25^ ^]^ For example, KLF9 could regulate adipogenesis via activating PPARγ2 promoter by interacting with C/EBPα.^[^ ^24^ ^]^ Furthermore, we have demonstrated KLF9 could direct bind to the promoter of *Pgc1a* thereby promote hepatic gluconeogenesis.^[^ ^25^ ^]^ An early study demonstrated that the ablation of KLF9 led to subfertility, uterine hypoplasia, and partial progesterone resistance in female mice.^[^ ^26^ ^]^ In addition, KLF9 could act as a transcriptional repressor of ERalpha, and the progesterone receptor and estrogen receptor influence epithelial cell proliferation.^[^ ^27^ ^]^ However, the role and mechanism by which KLF9 regulates PCOS in parenchymal cells of the liver are largely elusive.

Given the fact that PCOS is a complex metabolic disease and that the liver is the metabolic center of the body, we hypothesize that specific hepatic molecules affect the development of PCOS. In the present study, we identified KLF9 as a key molecule in hepatocytes. Intriguingly, hepatic KLF9 deficiency protected against DHEA‐induced PCOS, and hepatic KLF9 transgenic mice spontaneously exhibited a PCOS‐like phenotype. Furthermore, we revealed that intranuclear KLF9 directly binds to the promoter of *Srd5a1* and the gene loci of *Hsd3b3*. Our work documents a previously unrecognized conception that liver is a key extraovarian organ that affects the development of PCOS and that KLF9 is the crucial mediator of this process.

## 2 Results

### 2.1 KLF9 Expression is Increased in Hepatocytes from a Mouse Model of PCOS Induced by DHEA

Increasing evidence has demonstrated that patients with PCOS are more susceptible to nonalcoholic fatty liver disease (NAFLD), indicating that ovarian dysfunction leads to liver disorders.^[^ ^28^ ^]^ In contrast, whether the liver plays a role in the development of PCOS remains enigmatic. To investigate the role of the liver in the development of PCOS, we established a PCOS model in C57BL/6J mice using DHEA as previously described.^[^ ^29^ ^]^ Using vaginal cytology (Figure S1A, Supporting Information), we revealed that mice subjected to DHEA exhibited disrupted estrous cycles compared with those in the control group (Figure S1B, Supporting Information). Apart from poor fertility (Figure S1C, Supporting Information), mice subjected to DHEA displayed polycystic morphology accompanied with reduced corpora lutea number compared with those vehicle treatment (Figure S1D, Supporting Information). Similarly, the administration of DHEA led to a substantial elevated serum LH levels (Figure S1E, Supporting Information), decreased FSH levels (Figure S1F, Supporting Information), which causing a significant increase in circulating LH/FSH ratio (Figure S1G, Supporting Information) compared with mice treated with vehicles. In addition, DHEA‐treated mice displayed increase serum T and DHT levels (Figure S1H,I, Supporting Information), decreased glucose and insulin tolerance (Figure S1J,K, Supporting Information) in comparison with mice treated with vehicles. Collectively, these data were consistent with the features of DHEA‐induced PCOS, indicating that our model was established successfully.

**Figure 1:**
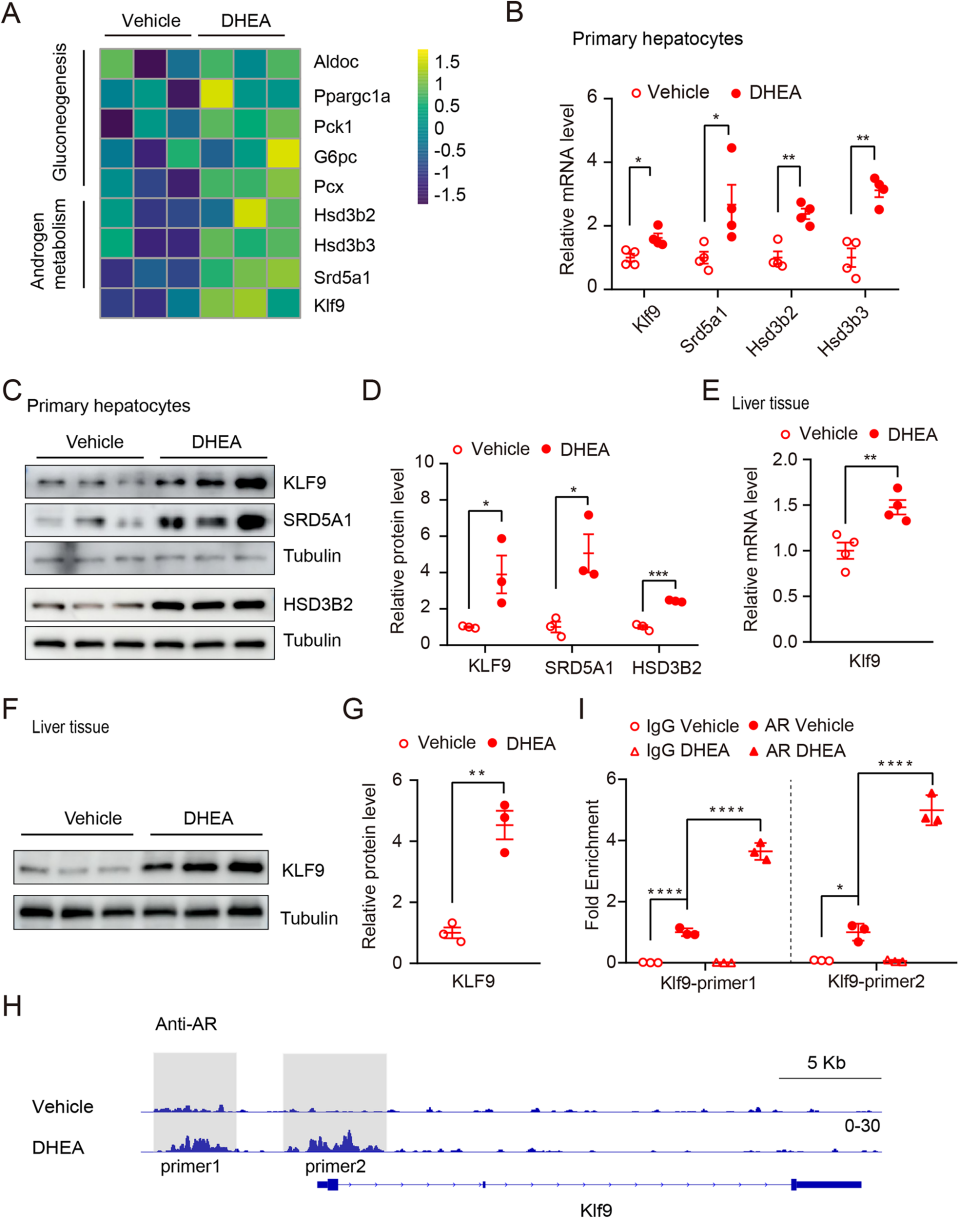
KLF9 is increased in hepatocytes of murine PCOS model induced by DHEA. A) Heatmap of differentially upregulated genes hepatocytes of mice administrated with DHEA or vehicle (n = 3 per group). B) Quantification mRNA expression of selective genes in hepatocytes of mice treated with DHEA or vehicles. C) Immunoblot analysis of KLF9, SRD5A1, and HSD3B2 expression in primary hepatocytes of mice treated with DHEA or vehicles. D) Quantification relative protein level of KLF9, SRD5A1, and HSD3B2 in (C). E) Quantification *Klf9* mRNA expression of selective genes in liver tissue of mice treated with DHEA or vehicles. F) Immunoblot analysis of KLF9 in primary liver tissue of mice treated with DHEA/vehicles. G) Quantification relative protein level of KLF9 in (F). H) IGV genome tracks showing AR enrichment at the *Klf9* promoter in primary hepatocytes. I) ChIP‐qPCR analysis of *Klf9* level on the gene body regions of AR. Data are presented as mean ±SD. Statistical analysis was performed using two‐tailed unpaired *t*‐tests (B, D) and Two‐way ANOVA(I). *p < 0.05, **p < 0.01, ***p < 0.001 and ****p < 0.0001.

In an attempt to identify the key mediators in the liver that might affect the development of DHEA‐induced PCOS in mice, we isolated primary hepatocytes from PCOS and control mice and performed bulk RNA‐seq (**Figure** **1**A). The data analysis indicated that the expression of glycogenesis‐related genes such as *Aldoc*, *Pparga1a*, *Pck1, G6pc*, and *Pcx* was upregulated, consistent with previous reports that PCOS mice exhibit an impaired glucose metabolism.^[^ ^30^ ^]^ Intriguingly, the expression of genes related to androgen metabolism, such as *Hsd3b2*, *Hsd3b3*, and *Srd5a1* was significantly upregulated. Notably, among the up‐regulated genes, *Klf9*, an important gene involved in glucose and lipid metabolism, was significantly increased after DHEA treatment. (Figure 1A). Our real‐time PCR and Western blot analyses confirmed that KLF9 expression was elevated in both primary hepatocytes and liver tissue at both the mRNA level and protein levels (Figure 1B–G), indicating that DHEA promotes KLF9 expression in hepatocytes of a PCOS mouse model. In addition, the expression of Srd5a1 and Hsd3b2 were increased in hepatocytes upon DHEA treatment both at mRNA and protein level (Figure 1B–D), which was in line with the result of RNA‐seq analysis (Figure 1A).

DHEA can be converted into DHT, which is the most biologically active endogenous androgen under a series of enzymatic reactions and DHT mainly exerts its function via AR.^[^ ^17^, ^31^, ^32^ ^]^ Once DHT binds to AR, the DHT/AR complex is translocated into the nucleus to mediate target gene expression. To identify potential AR target genes involved in androgen metabolism in hepatocytes, we administered DHEA to WT C57BL/6J mice to establish a PCOS model and then isolated primary hepatocytes to conduct a CUT&Tag assay. Sequence data analysis revealed that compared with that in the vehicle‐treated group, the binding of AR to the promoter of *Klf9* in the DHEA‐treated group was significantly greater (Figure 1H). In addition, the following ChIP‐qPCR analysis in primary hepatocytes further confirmed that DHEA‐treatment enhances the ability of AR binding to the *Klf9* promoter (Figure 1I). Therefore, KLF9 may play an important role in DHEA‐induced PCOS.

### 2.2 Hepatic Deletion of *Klf9* Ameliorated the Development of DHEA‐Induced PCOS

**Figure 2:**
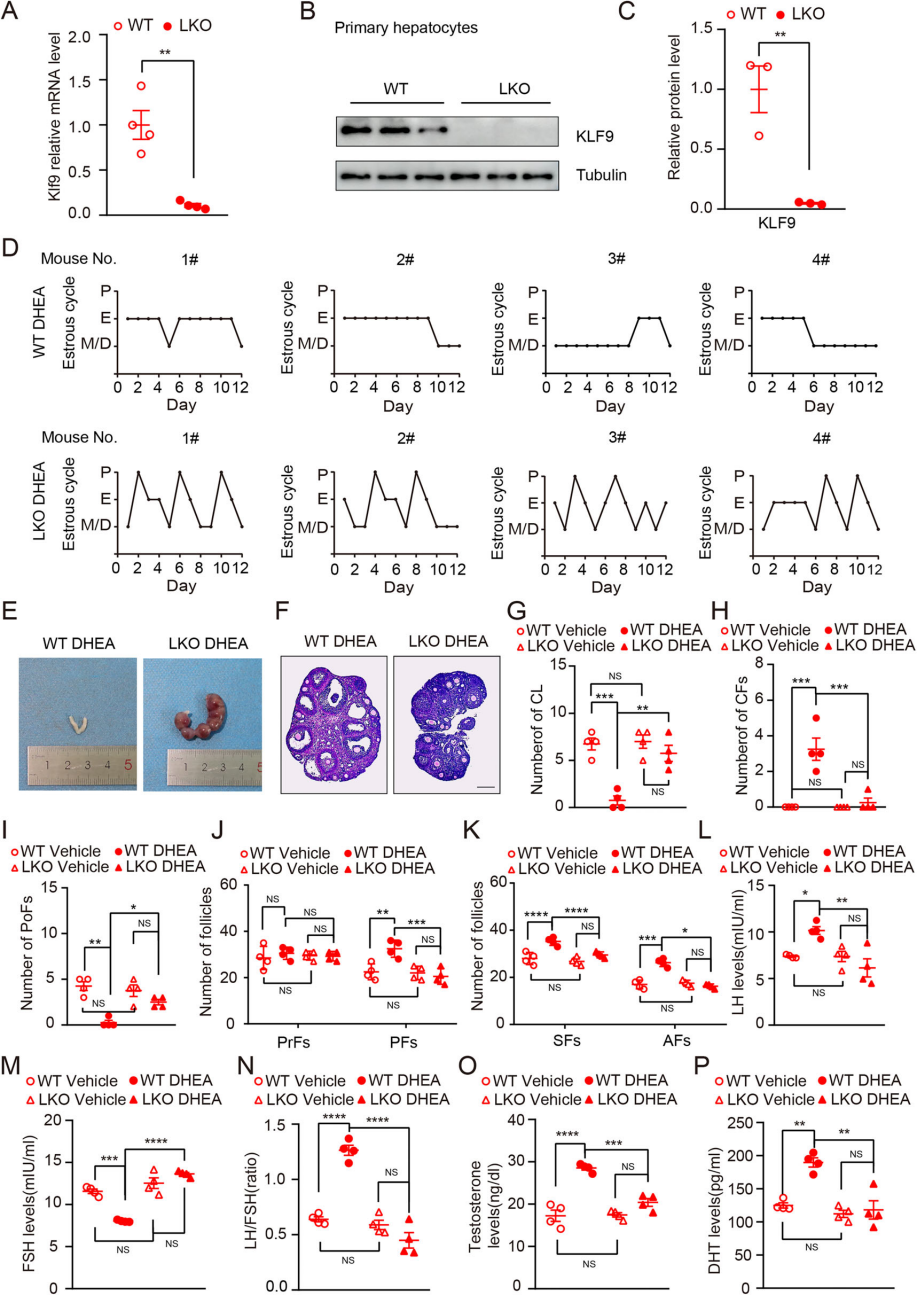
Hepatic Klf9 deficiency protects against DHEA induced PCOS. A) Quantification *Klf9* mRNA expression in liver tissue of WT and LKO mice (n = 4 per group). B) Immunoblot analysis of KLF9 expression in hepatocytes of WT and LKO mice. C) Quantitative analysis of KLF9 expression in (B). D) Representative estrous cyclicity of WT mice and LKO mice induced by DHEA (n = 4 per group). P, proestrus; E, estrus; M/D, metestrus/diestrus. E) Representative images of uterine implantation of LKO and WT female mice. F) Representative ovaries image of hematoxylin and eosin staining of WT mice and LKO mice, Scale bar: 200 µm. G,H) Quantification the number of corpora lutea (CL) and cystic follicles (CFs) in WT mice and LKO mice treated with or without DHEA (n = 4 per group); I–K) Quantification the number of different stage follicles in WT mice and LKO mice treated with or without DHEA (n = 4 per group); PoFs, Preovulatory follicles; PrFs, primordial follicles; PFs, primary follicles; SFs, secondary follicles; AFs, antral follicles. L–N) Serum levels of LH (L), FSH(M), and the ratio of LH/FSH(N) of WT mice and LKO mice treated with or without DHEA (n = 4 per group). O,P) Serum levels of testosterone(O) and DHT(P) of WT mice and LKO mice treated with or without DHEA (n = 4 per group). Data are presented as mean ±SD. Statistical analysis was performed using two‐tailed unpaired *t*‐tests (A, C) and Two‐way ANOVA (G‐P). *p < 0.05, **p < 0.01, ***p < 0.001 and ****p < 0.0001.

To explore the role of hepatic KLF9 in the development of PCOS in vivo, we generated a hepatocytes‐specific *Klf9* knockout mouse model (*Alb‐Cre^+^*; *Klf9* ^fl/fl^, hereafter referred to as LKO) by crossing mice carrying the floxed *Klf9* allele ^[^ ^25^ ^]^ with mice expressing Cre recombinase under the control of the *albumin* promoter and enhancer, littermates lacking the Cre gene were used as controls (*Alb‐Cre^−^*; *Klf9* ^fl/fl^, hereafter referred to as WT). Real‐time PCR and Western blot analyses confirmed the absence of KLF9 in the liver and hepatocytes of LKO mice respectively (**Figure** **2**A–C).

After establishing the PCOS model in LKO and WT mice by the daily injection of DHEA for 21 days, we assessed ovarian function in the subsequent experiments. As evidenced by vaginal epithelial cell smears, *Klf9* deletion in hepatocytes resulted in a better estrous cycle under PCOS conditions (Figure 2D). To assess fertility, WT and LKO mice with PCOS were mated with WT C57BL/6J male mice, and the results showed that LKO mice with PCOS had better fertility than did WT mice with PCOS (Figure 2E; Figure S2A, Supporting Information). To further investigate whether KLF9 affects the DHEA‐induced PCOS phenotype, we conducted the following experiments. H&E staining of ovaries revealed that *Klf9* deletion in hepatocytes markedly improved ovarian morphology in the setting of PCOS condition, such as increased corpora lutea number and reduced cystic follicles (Figure 2F–H). Furthermore, follicles counting showed that LKO mice subjected to DHEA exhibited an increased number of preovulatory follicles, while fewer primary follicles, secondary follicles and antral follicles relative to WT mice subjected to DHEA (Figure 2I–K), suggesting that hepatic *Klf9* deletion ameliorates DHEA‐induced ovulatory abnormalities. Additionally, ELISAs revealed that KLF9 deficiency markedly improved each of the indicators including LH, FSH, LH/FSH, T and DHT in the serum than that of littermates (Figure 2L–P). Overall, these results provide evidence that hepatic *Klf9* deficiency protects against the development of PCOS induced by DHEA.

### 2.3 Hepatic KLF9 Deletion Inhibited the Expression of Androgen Conversion‐Related Enzymes in the Liver and Ameliorated Glucose Intolerance Induced by Treatment with DHEA

**Figure 3:**
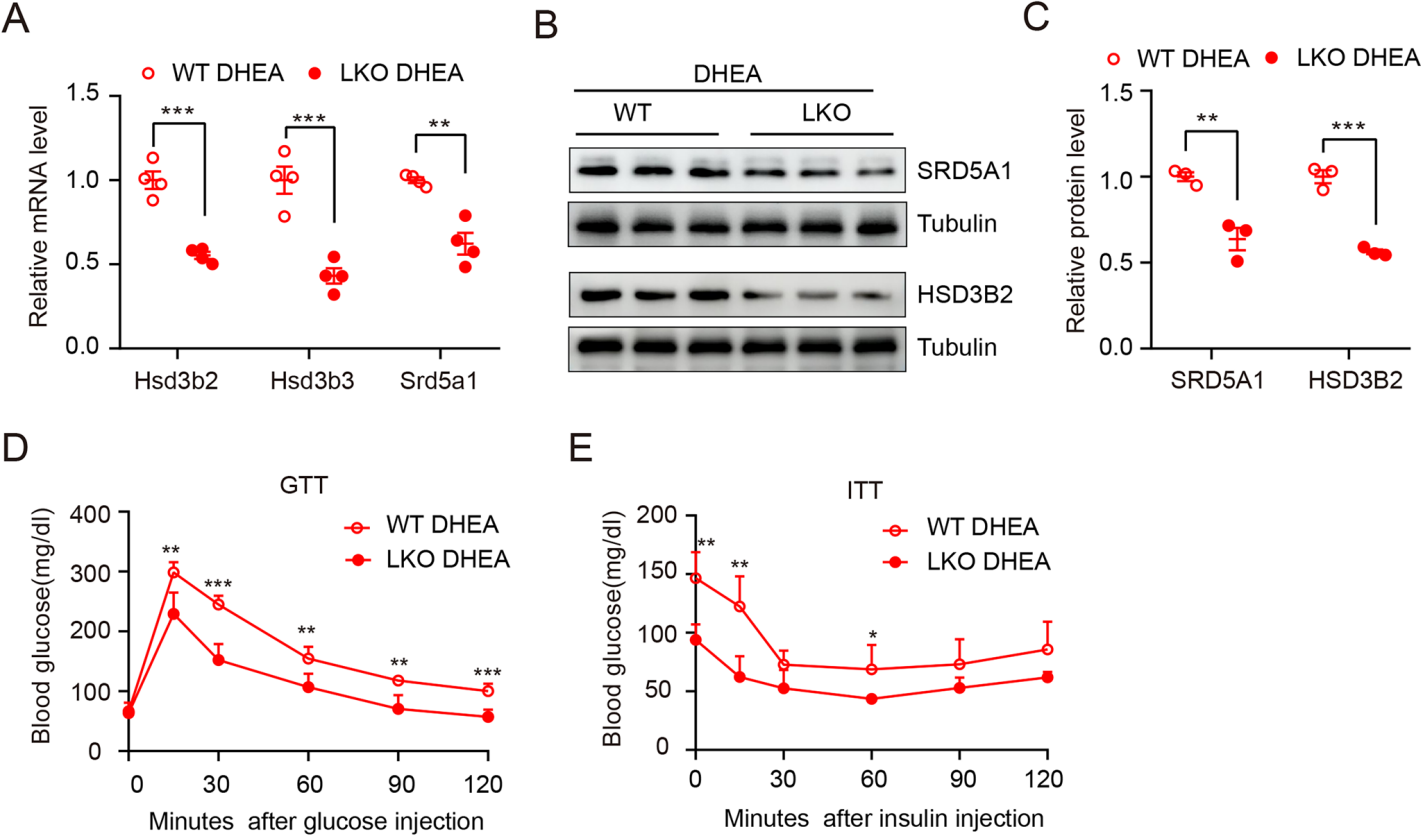
Hepatic KLF9 deletion inhibited the expression of androgen conversion‐related enzymes in the liver. A) Quantification mRNA expression of genes related to androgen conversion relative to *36b4* (n = 4 per group). B) Immunoblot analysis of SRD5A1 and HSD3B2 in liver of WT mice and LKO mice. C) Quantification relative protein level of SRD5A1 and HSD3B2 in (B). D,E) Glucose tolerance test and insulin tolerance test results (n = 4 per group). Data are presented as mean ±SD. Statistical analysis was performed using two‐tailed unpaired *t*‐tests (A,C–E). *p < 0.05, **p < 0.01 and ***p < 0.001.

We observed that hepatic *Klf9* deletion led to a dramatically decreased circulating LH/FSH ratio and decreased T and DHT levels, suggesting KLF9 might regulate key enzyme(s) involved in androgen conversion in the liver. To test this hypothesis, we examined androgen conversion‐related enzyme expression in the liver. The ablation of *Klf9* in hepatocytes decreased the expression of androgen conversion‐related enzymes expression, such as *Hsd3b2*, *Hsd3b3* and *Srd5a1*, in liver tissue (**Figure** **3**A). Since SRD5A1 and HSD3B2 are key enzymes that catalyzes the conversion of DHEA to DHT,^[^ ^33^, ^34^ ^]^ we further assessed the expression of both proteins in liver tissue. Western blot analysis revealed that the SRD5A1 and HSD3B2 level in the liver tissue of LKO mice was significantly lower than that in the liver tissue of WT mice (Figure 3B,C).

As insulin resistance and glucose metabolism dysregulation are complications of PCOS, we assessed the levels of GTT and ITT in WT and LKO mice. Compared with their control littermates, LKO mice had improved glucose tolerance and insulin sensitivity (Figure 3D,E), finding that they were consistent with other symptoms of PCOS in LKO mice. Additionally, improvements in glucose tolerance and insulin sensitivity in LKO female mice were consistent with those in male mice.^[^ ^25^ ^]^ Collectively, these data indicate that KLF9 deletion results in the decreased expression of androgen conversion‐related enzymes, especially Srd5a1 and Hsd3b3.

### 2.4 Hepatic KLF9 Transgenic Mice Exhibited a Spontaneous PCOS‐Like Phenotype

**Figure 4:**
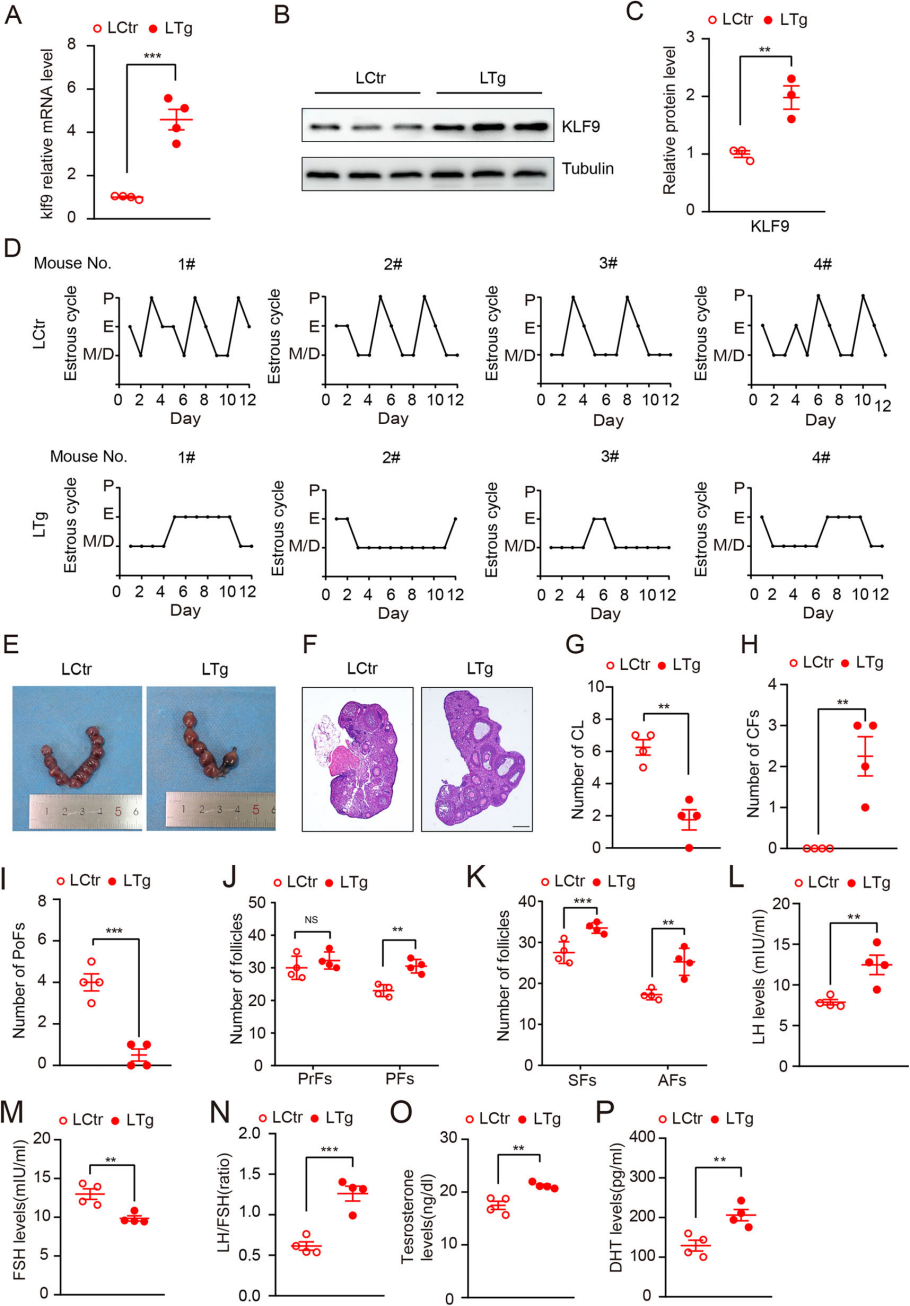
Liver‐specific KLF9 transgenic mouse display a spontaneously PCOS phenotype. A) Quantification mRNA expression of *Klf9* expression in liver tissue of LCtr mice and LTg mice (n = 4 per group). B) Immunoblot analysis of KLF9 expression in liver tissue of LCtr mice and LTg mice. C) Quantification relative protein level of KLF9 in (B). D) Representative estrous cyclicity of LCtr mice and LTg mice (n = 4 per group). P, proestrus; E, estrus; M/D, metestrus/diestrus. E) Representative images of uterine implantation of LCtr mice and LTg mice. F) Representative ovaries image of hematoxylin and eosin staining of LCtr mice and LTg mice, Scale bar: 200 µm. G,H) Quantification the number of corpora lutea (CL) and cystic follicles (CFs) in LCtr mice and LTg mice (n = 4 per group); I–K) Quantification the number of different stage follicles in the ovary of LCtr mice and LTg mice (n = 4 per group). CFs, cystic follicles; PoFs, Preovulatory follicles; PrFs, primordial follicles; PFs, primary follicles; SFs, secondary follicles; AFs, antral follicles. L–N) Serum levels of LH(L), FSH(M), and the ratio of LH/FSH(N) of LCtr mice and LTg mice (n = 4 per group). O,P) Serum levels of testosterone(O) and DHT(P) of LCtr mice and LTg mice (n = 4 per group). Data are presented as mean ±SD. Statistical analysis was performed using two‐tailed unpaired *t*‐tests (A, C, G‐P). *p < 0.05, **p < 0.01and ***p < 0.001.

In a previous study, we generated Rosa26 *Klf9^fl/fl^* mice ^[^ ^35^ ^]^ utilizing the CRISPR/Cas9 system to insert the CAG‐LoxP‐STOP‐LoxP‐Klf9 cassette into the mouse Rosa26 locus to allow *Klf9* expression in the presence of Cre recombinase. In the present study, to further verify the role of hepatic KLF9 in the development of PCOS, we crossed Rosa26 *Klf9^fl/fl^* mice with *Alb‐cre* mice to generate hepatocyte‐specific *Klf9* transgenic mice (*Alb‐Cre^+^*; Rosa26 *Klf9^fl/+^*, hereafter named LTg); littermates lacking the Cre gene were used as controls (*Alb‐Cre^−^*; Rosa26 *Klf9^fl/+^*, hereafter named LCtr). Importantly, we could not obtain homozygote hepatic *Klf9* transgenic mice, possibly due to the poor fertility. Real‐time PCR and Western blot analysis confirmed that KLF9 was increased specifically in the liver of LTg mice compared with that in the livers of LCtr mice (**Figure** **4**A–C).

Notably, we observed that the number of pups per litter produced by *Klf9* transgenic mice was significantly lower than that produced by littermates (data not shown), suggesting that hepatic *Klf9* gain of function has an impact on the productive system. Thus, we assessed ovarian function of LTg and LCtr mice in the following experiments. In contrast to the phenotype of LKO mice, vaginal cytology revealed that the estrous cycling was disrupted in LTg mice compared with that in LCtr mice under normal conditions (Figure 4D). We then mated LTg female mice and their littermates with WT C57BL/6J male mice, and the results showed that LTg mice had fewer embryos than did LCtr mice (Figure 4E; Figure S2B, Supporting Information). In addition, H&E staining revealed that the ovaries of LTg mice had PCOS‐like morphology, such as reduced corpora lutea number and increased cystic follicles (Figure 4F–H). Follicles counting analysis showed that LTg mice had more primary follicles, secondary follicles and antral follicles, while fewer preovulatory follicles than did those of LCtr mice (Figure 4I–K), suggesting *Klf9* overexpression in hepatocytes evokes abnormal ovulation in mice. We further measured the levels of serum hormones in the mice. ELISAs revealed that *Klf9* mutation led to elevated LH level, decreased FSH level, which causing a significant increase in LH/FSH ratio (Figure 4L–N). Moreover, ELISAs displayed that the serum T and DHT were greater in LTg mice than in LCtr mice (Figure 4O,P), which was phenotypically opposite to the findings in LKO mice. Thus, the results of the above experiments suggest that the gain of function of *Klf9* in hepatocytes leads to the development of PCOS in vivo.

### 2.5 The Gain of Hepatic KLF9 Function Promoted Expression of Androgen Conversion‐Related Enzymes in the Liver and Led to Impaired Glucose Metabolism

**Figure 5:**
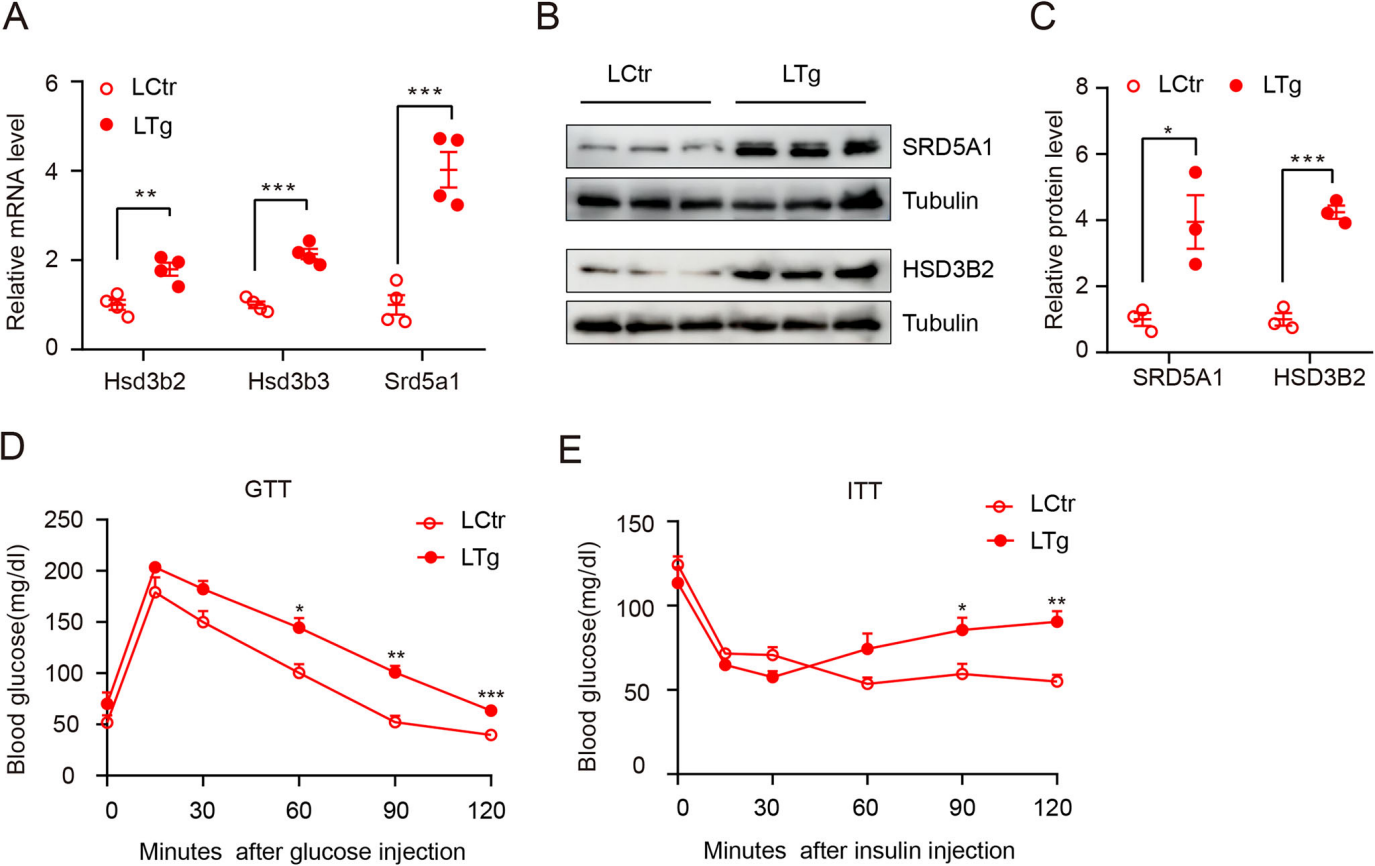
Hepatic KLF9 transgenic promotes the expression of androgen conversion‐related enzymes in the liver. A) Quantification mRNA expression of androgen conversion‐related genes relative to *36b4* in liver of LCtr mice and LTg mice (n = 4 per group). B) Immunoblot analysis of SRD5A1 and HSD3B2 in liver of LCtr mice and LTg mice. C) Quantification relative protein level of SRD5A1 and HSD3B2 in (B). D,E) Glucose tolerance test and insulin tolerance test results of LCtr mice and LTg mice (n = 4 per group). Data are presented as mean ±SD. Statistical analysis was performed using two‐tailed unpaired *t*‐tests (A, C, D,E). *p < 0.05, **p < 0.01 and ***p < 0.001.

A number of studies have shown that PCOS may contribute to metabolic disorders, including insulin resistance ^[^ ^30^, ^36^ ^]^ and NASH,^[^ ^28^ ^]^ suggesting that the ovary plays a role in liver function in PCOS. However, whether the liver affects the pathological process in the setting of PCOS remains largely unclear. Since we demonstrated that *Klf9* deletion in hepatocytes led to the decreased expression of SRD5A1, an androgen conversion‐related enzyme, we next wanted to determine whether this phenotype could be reconstituted in the livers of hepatic KLF9 transgenic mice. The hepatic *Klf9* transgene significantly increased the androgen conversion‐related genes, such as *Hsd3b2*, *Hsd3b3* and *Srd5a1*, at the mRNA level (**Figure** **5**A). Moreover, Western blot analysis further showed that SRD5A1 and HSD3B2 expression was markedly increased in the liver tissue of hepatic KLF9 transgenic mice (Figure 5B,C), suggesting that the hepatic *Klf9* transgene promotes androgen conversion in the liver. In contrast to GTT and ITT results for LKO mice, LTg mice displayed a impaired glucose tolerance and impaired insulin tolerance than did LCtr mice (Figure 5D,E), which is akin to the phenotype of male mice with hepatic adenoviral *Klf9* overexpression.^[^ ^25^ ^]^ Collectively, these results demonstrate that genetic *Klf9* expression contributes to the expression of androgen conversion‐related enzymes in the liver.

### 2.6 DHEA‐Induced KLF9 was Essential for Androgen Conversion in Hepatocytes

**Figure 6:**
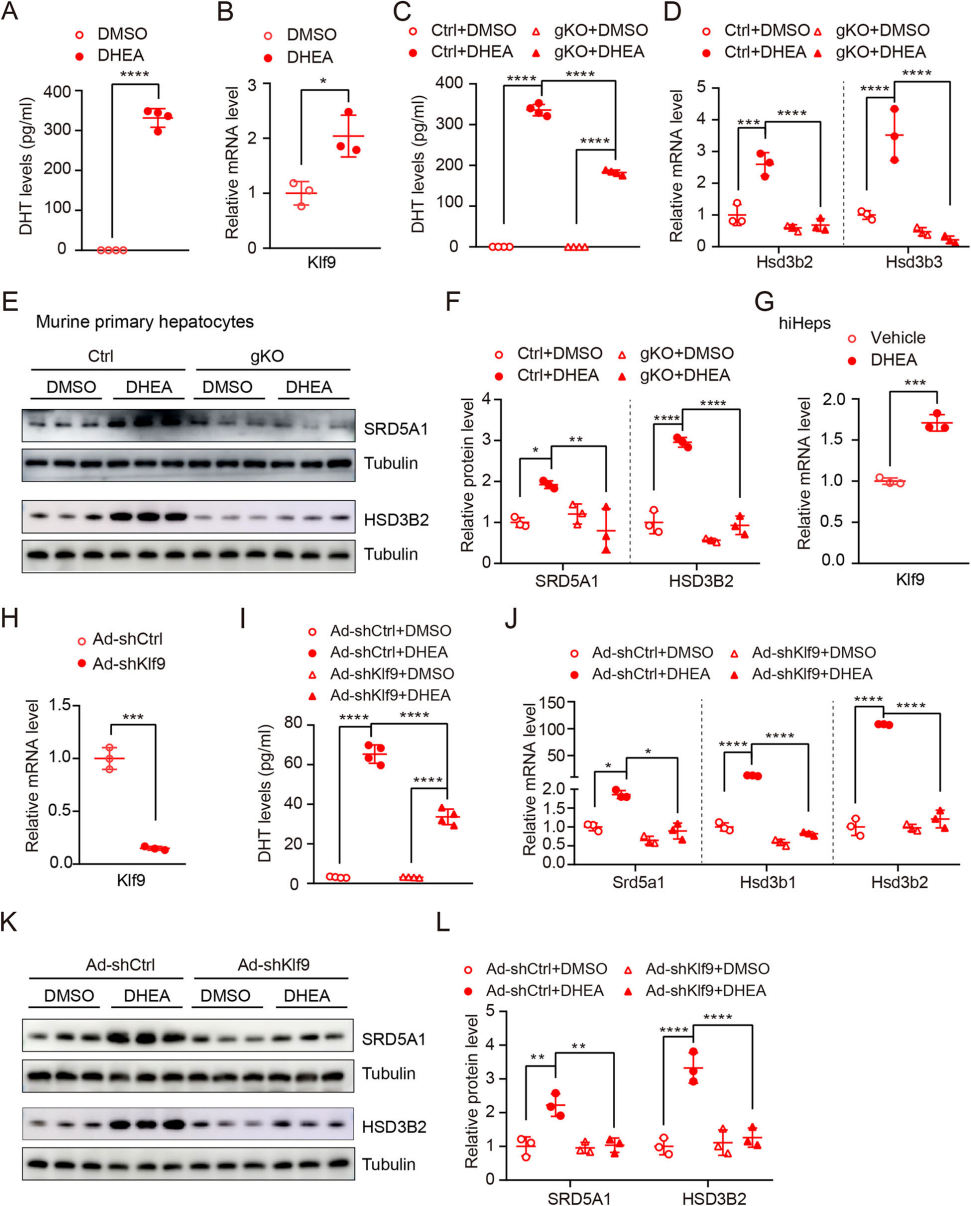
KLF9 is essential for DHEA to T and DHT conversion in hepatocytes in vitro. A) ELISA‐based quantification of DHT level in the supernatants of murine primary hepatocytes treated with DMSO (control) and DHEA (20 µM) for 24 h. B) Quantification *Klf9* mRNA expression in murine primary hepatocytes treated with DMSO (control) and DHEA (20 µM) for 24 h. C) ELISA‐based quantification of DHT level in the supernatants of isolated primary hepatocytes from gKO and Ctrl mice treated with DMSO (control) and DHEA (20 µM) for 24 h. D) Quantification mRNA expression of androgen conversion‐related genes relative to *36b4* of isolated primary hepatocytes from gKO and Ctrl mice treated with DMSO (control) and DHEA (20 µM) for 24 h. E) Immunoblot analysis of SRD5A1 and HSD3B2 expression in isolated primary hepatocytes from gKO and Ctrl mice treated with DMSO (control) and DHEA (20 µM) for 24 h. F) Quantification SRD5A1 and HSD3B2 protein expression in (E). G) Quantification *Klf9* mRNA expression in hiHeps with DMSO (control) and DHEA (20µM) for 24 h. H) Quantification *Klf9* mRNA expression in hiHeps after Ad‐shCtrl and Ad‐shKlf9 for 24 h. I) ELISA detection of DHT level in the supernatants of adenovirus‐mediated Klf9 knockdown hiHeps treated with DMSO (control) and DHEA (20 µM) for 24 h. J) Quantification androgen conversion‐related gene expression in adenovirus‐mediated *Klf9* knockdown hiHeps treated with DMSO (control) and DHEA (20 µM) for 24 h. K) Immunoblot analysis of SRD5A1 and HSD3B2 expression in adenovirus‐mediated *Klf9* knockdown hiHeps treated with DMSO (control) and DHEA (20 µM) for 24 h. L) Quantification SRD5A1 and HSD3B2 protein expression in (K). Data are presented as mean ±SD. Statistical analysis was performed using two‐tailed unpaired *t*‐tests (A, B, G, H) and Two‐way ANOVA (C,D, F, I,J). *p < 0.05, **p < 0.01, ***p < 0.001 and ****p < 0.0001.

Our previous data provide evidence that hepatic KLF9 promotes androgen production in the context of PCOS, but whether this occurs directly remains unclear. In this scenario, we assessed whether KLF9 is required for direct androgen conversion in hepatocytes. For this purpose, we isolated WT mouse hepatocytes and treated the cells with 20 µM DHEA for 48 h. ELISAs revealed a significant increase in DHT in the medium supernatant of primary hepatocytes after the DHEA treatment (**Figure** **6**A). Real‐time PCR analysis indicated that exposure to DHEA promoted the expression of *Klf9* (Figure 6B). To determine KLF9 is required for the conversion of DHEA to DHT in hepatocytes, primary hepatocytes were isolated from the livers of global *Klf9*‐mutant mice (gKO) and littermates (Ctrl),^[^ ^25^ ^]^ followed by treatment with DHEA or DMSO. ELISAs revealed that the treatment of Ctrl primary hepatocytes with DHEA markedly increased cellular DHT synthesis, whereas *Klf9* mutation significantly attenuated the synthesis of this androgen (Figure 6C). In addition, real‐time PCR analysis showed that DHEA significantly increased the expression of androgen conversion‐related genes in Ctrl primary hepatocytes such as *Hsd3b2* and *Hsd3b3*, whereas *Klf9* mutation reduced the DHEA‐mediated induction these gene expression (Figure 6D). Western blot analysis exhibited that DHEA treatment led to an upregulation of SRD5A1 and HSD3B2, whereas *Klf9* mutation significantly attenuated the elevation of SRD5A1 and HSD3B2 (Figure 6E,F). These data suggest an essential role of KLF9 in mediating the conversion of DHEA to DHT.

Next, we assessed whether the same effect exists in human hepatocytes. Since we could not obtain human primary hepatocytes, human induced hepatocytes (hiHeps) were used in this study as previously reported.^[^ ^37^ ^]^ Similar to the murine primary hepatocytes, DHEA treatment significantly increased *Klf9* expression in hiHeps (Figure 6G). Then, we prepared adenoviruses for *Klf9* expression interference. Real‐time PCR analysis showed that the adenoviruses worked well (Figure 6H). After infection with adenovirus for 24 h, hiHeps were subsequently treated with 20 µM DHEA for another 24 h. As expected, *Klf9* knockdown by adenovirus (Ad‐shKlf9) resulted in in markedly decreased DHT in the medium supernatant of hiHeps after treatment with DHEA (Figure 6I). As mouse *Hsd3b3* is homologous to the human *Hsd3b1* and *Hsd3b2*, we detected the expression of *Hsd3b1* and *Hsd3b2* in hiHeps. Knockdown of *Klf9* in hiHeps significantly abolished the expression of Hsd3b2 and Srd5a1 after DHEA treatment both at mRNA and protein level (Figure 6J–L). Taken together, these results demonstrate that androgen conversion occurs in hepatocytes and that KLF9 is essential for this process.

### 2.7 KLF9 Directly Promoted the Expression of Androgen Conversion‐ Related Enzymes in Hepatocytes

**Figure 7:**
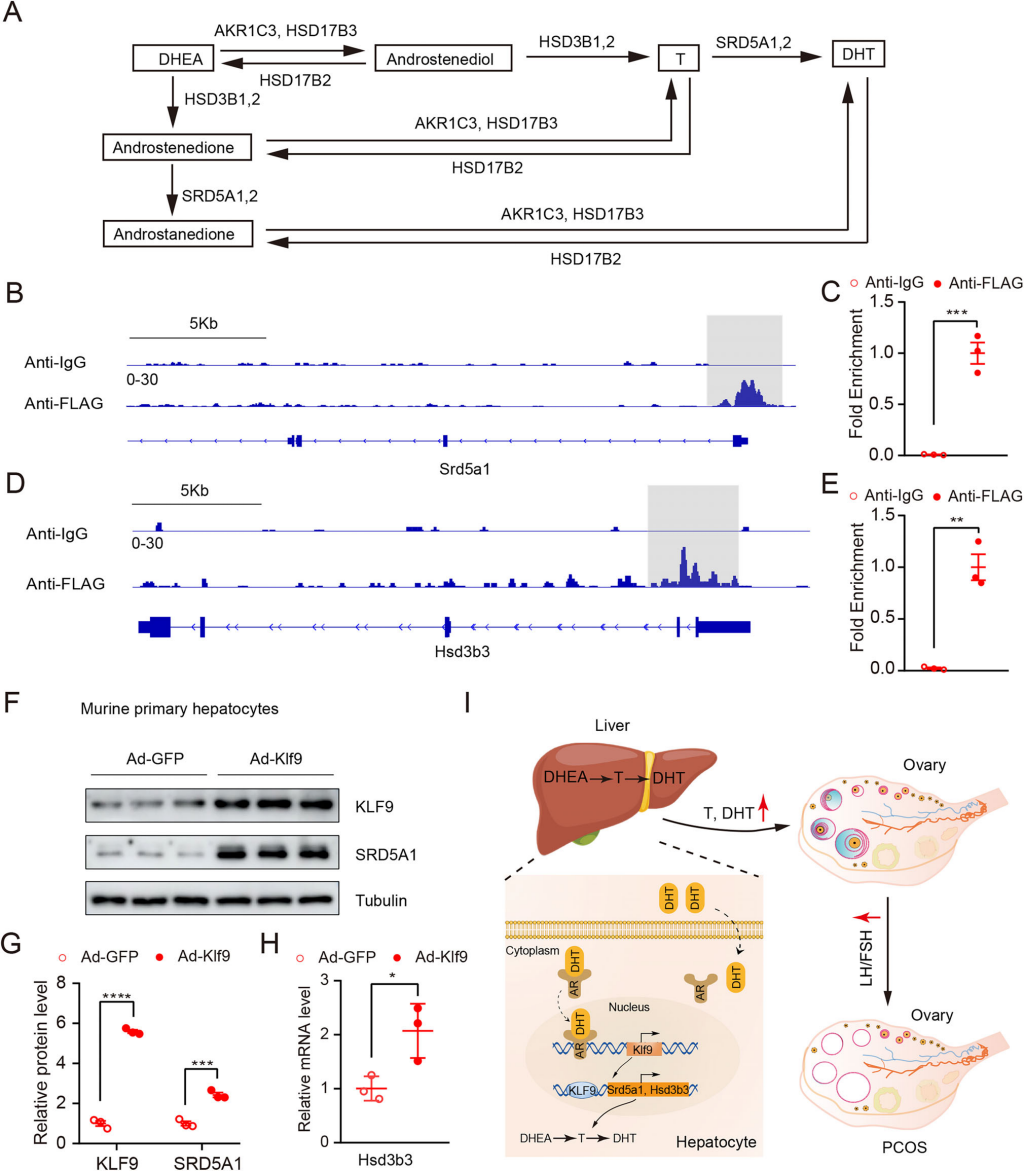
KLF9 directly targets *Srd5a1* and *Hsd3b3*. A) Schematic diagram showing the androgen conversion process. B) IGV genome tracks showing KLF9 enrichment at the promoter of *Srd5a1* in primary hepatocytes. C) ChIP‐qPCR analysis of *Srd5a1* fold enrichment in Flag IP. D) IGV genome tracks showing KLF9 enrichment at the gene loci of *Hsd3b3* in primary hepatocytes. E) ChIP‐qPCR analysis of *Hsd3b3* fold enrichment in Flag IP. F) Immunoblot analysis the expression of SRD5A1 in isolated primary hepatocytes after overexpression of Klf9 mediated by adenovirus for 24 h; G) Quantification protein levels of KLF9 and SRD5A1 in (F). H) Quantification *Hsd3b3* mRNA expression in isolated primary hepatocytes after overexpression of *Klf9* mediated by adenovirus for 24 h. I) Proposed model of hepatic KLF9 aggravating the development of PCOS. In hepatocytes, after a series of reaction, DHEA converted into DHT, which binds to AR in the cytosol. Then, the DHT/AR complex translocated into the nucleus to activate *Klf9* transcription, which further promotes the transcription of *Srd5a1* and *Hsd3b 3*. Data are presented as mean ±SD. Statistical analysis was performed using two‐tailed unpaired *t*‐tests (C, E, G, H). *p < 0.05, **p < 0.01, ***p < 0.001 and ****p < 0.0001.

After a series of enzymatic reactions, DHEA is converted into the active androgen T, which is further converted into a more potent form, DHT, in the presence of SRD5A1/SRD5A2^[^ ^31^, ^38^ ^]^(**Figure** **7**A). As a transcription factor belonging to the KLF family, *Klf9* alters the expression of its target genes, mainly at the transcription level.^[^ ^22^ ^]^ Our aforementioned data indicate that KLF9 is a target gene for AR and that KLF9 is required for the androgen conversion in hepatocytes. Considering the importance of androgen conversion in the development of PCOS, we speculate that KLF9 might regulate the critical genes encoding enzymes responsible for androgen conversion. Owing to the lack of available primary antibody of KLF9 for ChIP‐seq, we generated 3×FLAG‐EGFP‐CreERT2 constitutive knock‐in mice (hereafter referred to as 3×FLAG‐KLF9 KI) using CRISPR‐Cas9 technology. This mouse model allowed us to measure and immunoprecipitated KLF9 by using anti‐FLAG antibodies, detect KLF9 expression based on EGFP and characterize and trace KLF9‐expressing cells spatiotemporally after tamoxifen administration. To construct the targeting vector, the *Klf9* gene with a 3×FLAG cassette was inserted between the last coding exon and the 3′UTR of *Klf9* (Figure S3A, Supporting Information). Genotyping PCR revealed that the cassette sequence was inserted correctly (Figure S3B, Supporting Information) and Western blot analysis confirmed the successful knock‐in of FLAG (Figure S3C, Supporting Information). To test our hypothesis, we isolated primary hepatocytes from 3×FLAG‐KLF9 KI PCOS mice and performed a CUT&Tag assay. Intriguingly, we noticed that KLF9 directly binds to the promoter of *Srd5a1* and the gene loci of *Hsd3b3* (Figure 7B,D). ChIP‐qPCR analysis further confirmed these results (Figure 7C,E). To explore whether *Klf9* promotes androgen conversion‐related enzyme expression, we generated an adenovirus expressing Klf9 (Ad‐Klf9) and performed real‐time PCR and Western blot analyses of primary hepatocytes infected with Ad‐Klf9. The data confirmed that the induction of Klf9 expression by Ad‐Klf9 in primary hepatocytes strongly activated *Srd5a1* and *Hsd3b3* expression (Figure 7F–H).

Based on present data in this study, we developed the following model for the role and mechanism of KLF9 in the development of PCOS (Figure 7I). DHEA can be converted to more potent DHT in hepatocytes in the presence of various enzymes. The binding of DHT to cytoplasmic AR results in its nuclear translocation and binding to the promoter of *Klf9* to promote *Klf9* transcription, which further drives the expression of *Srd5a1* and *Hsd3b3* to synthesize DHT. In brief, DHEA can be converted to more potent DHT in hepatocytes through the DHT/AR/KLF9/SRD5A1&HSD3B3 androgen conversion enzyme signaling pathway. During this process, KLF9 serves as an accelerator of DHT synthesis.

## 3 Discussion

The pathogenesis of PCOS is mainly based on the theory of the hypothalamic‐pituitary‐gonadal axis. Gonadotropin releasing hormone (GnRH) leads to luteinizing hormone (LH) secretion, which further stimulates the ovarian theca cells to produce androgen.^[^ ^39^ ^]^ The traditional theory suggests that ovarian theca cells are the main cell type that produces excess androgen during the process of PCOS.^[^ ^5^ ^]^ However, whether hepatocytes are involved in androgen production remains largely unexplored. In addition, previous studies have focused on the effects of PCOS on liver function. For instance, Cui P et al. reported that long‐term androgen excess induces nonalcoholic fatty liver disease in a PCOS‐like rat model.^[^ ^5^, ^39^ ^]^ However, whether the liver plays a role in PCOS and the mechanisms have not been investigated. In our study, we revealed that hepatocytes play a pivotal role in maintaining androgen metabolic homeostasis. Our work reveals the role of the liver‐ovary axis in the development of PCOS.

Hyperandrogenism is an important clinical characteristic of PCOS. Under normal physiological circumstances, androgens are mainly produced by the adrenal cortex and theca cells of the ovary.^[^ ^40^ ^]^ In PCOS patients with androgen excess, the primary source of androgens is the theca cells of the ovary.^[^ ^41^, ^42^, ^43^ ^]^ According to available knowledge, the metabolic processes of androgens occur mainly in the adrenal glands and ovaries. However, whether androgen conversion occurs in hepatocytes remains largely unclear. In the present study, ELISAs displayed significant elevated levels of testosterone and dihydrotestosterone in primary hepatocytes after treatment with DHEA, indicating that the enzymatic reactions involving androgen occur in hepatocytes.

Recent reports have shown that extraovarian tissues affect the development of PCOS.^[^ ^44^ ^]^ For instance, brown adipose tissue transplantation was shown to alleviate the progression of DHEA‐induced PCOS rat.^[^ ^45^ ^]^ In addition, Aimee S. L et al. reported that the brain is a key site in the development of PCOS.^[^ ^47^ ^]^ Here, our study provides new evidence that extraovarian tissues play an important role in the development of PCOS, especially excess DHEA‐induced PCOS. It will be interesting to investigate whether tissues in addition to the brain, brown adipose tissue and liver that influence PCOS development.

Androgens function through the androgen receptor (AR), which is widely expressed in various cell types, including hepatocytes. AR signaling is important for the development of PCOS. Global AR knockout mice are resistant to androgen‐induced PCOS.^[^ ^47^ ^]^ The AR antagonist flutamide could be used to alleviate symptoms of PCOS in some patients.^[^ ^46^ ^]^ In the setting of PCOS, *Klf9* liver‐specific knockout mice display alleviated PCOS symptoms, a finding that is in consistent with the phenotype of AR global knockout mice. Our mechanistic experiment revealed that AR signaling promotes the expression of *Klf9*, which accelerates the androgen conversion by regulating key enzymes responsible for the conversion of T to DHT. Thus, anrogen‐AR‐KLF9 forms a positive feedback loop in hepatocytes to disrupt androgen homeostasis and accelerate PCOS progression. Previous studies have demonstrated that KLF9 is a target gene of the glucocorticoid (GC) receptor ^[^ ^25^, ^47^ ^]^ and thyroid hormone receptor ^[^ ^48^, ^49^ ^]^ and noradrenaline (NE) signaling pathways.^[^ ^50^ ^]^ KLF9 functions as a crucial intermediary gene in the biological functions of glucocorticoids, thyroid hormones and noradrenaline. However, whether AR targets KLF9 remains unclear. Here, by employing a CUT&Tag assay, we showed that AR directly binds the *Klf9* promoter, suggesting that KLF9 is a downstream target gene of AR. Therefore, our studies expand the understanding of the biological function of KLF9, especially in the liver.^[^ ^51^ ^]^

However, there are several limitations to this study. First, tracking the dynamic changes of liver‐oriented DHT in circulation is a big challenge in vivo. How to effectively quantify DHT produced by the liver AR/KLF9/SRD5A1&HSD3B pathway is very important for this study. Therefore, developing effective methods to solve these problems will be much appreciated. Second, for molecular mechanism study, considering the spatio‐temporal characteristics of transcriptional regulation in hepatocytes under PCOS conditions, we only showed CUT&Tag and ChIP‐qPCR data in isolated hepatocytes without cell lines in vitro study. Some data from hepatocytes in vivo and cell lines in vitro may be different due to the complexity of transcription factor regulation in a spatio‐temporal background.

In summary, we demonstrated that hepatic KLF9 affects the development of PCOS through the AR/KLF9/SRD5A1&HSD3B3 pathway. During this process, KLF9 serves as an accelerator of androgen production. In the setting of PCOS, hepatic KLF9 facilitates androgen synthesis by accelerating the conversion of DHEA to DHT in hepatocytes through Srd5a1and Hsd3b3, leading to PCOS progression. Nevertheless, our work provides evidence that hepatic KLF9 might be a therapeutic target for the treatment of androgen excess‐induced PCOS (for example, by adeno‐associated virus system ^[^ ^52^, ^53^ ^]^), revealing an unrecognized important role of the liver in the development of PCOS.

## 4 Experimental Section

### Animals Care and Ethical Approvals

All mice in experiments were bred and maintained under specific pathogen‐free conditions at Ningxia Medical University of Laboratory Animal Centre. Besides, all mice with ad libitum access to a standard chow diet and water in individually ventilated cages. *Klf9^fl/fl^*, Rosa26 *Klf9^fl/fl^*, Global *Klf9*‐mutant mice and *Alb‐cre* mice on C57BL/6J background were provide by prof. Yongsheng Chang from Tianjin Medical University. All mice were randomly assigned to each group, and the numbers in group as indicated. All studies involving mice were approved by Ethics Committee of Ningxia Medical University (Approval Number: 2023–035). Genotyping was performed as previously described.^[^ ^25^, ^35^ ^]^

### Generation of 3×FLAG‐KLF9 Constitutive Knock‐In Mouse

The 3×FLAG‐KLF9 constitutive knock‐in mouse on C57BL/6J background was generated using CRISPR/Cas9 methods in Beijing Biocytogen Co., Ltd. To this end, a targeting vector containing the *Klf9* gene (NM_01 0638.5) with a 3×FLAG cassette inserted between the last coding exon and 3′UTR of *Klf9* was constructed. After confirming the vector is functional, the targeting vector and sgRNA together with Cas9 mRNA were co‐microinjected into C57BL/6J zygotes, which were then implanted into the oviducts of Kunming pseudo‐pregnant surrogate mothers. Germline transmission was obtained through backcrossing chimeric offspring with C57BL/6J mice and detected by PCR, Sanger sequencing and Southern blot via tail genomic DNA.

### PCOS Model

PCOS mouse model was constructed as previously described.^[^ ^29^ ^]^ Briefly, DHEA (D4000, Sigma–Aldrich) were dissolved in corn oil and subcutaneously injected in 3‐week‐old female mice daily for 21 days. The PCOS model was confirmed by assessment of estrous cycle together with a glucose tolerance test (GTT) and insulin tolerance test (ITT).

### Assessment of Estrous Cycle

For determination of estrous cycle, vaginal epithelial cell smears were taken daily and subjected to H&E staining. Estrous‐cycle stage was determined based on cell type by microscopic analysis. Proestrus was characterized by rounded nucleated epithelial cells; estrus was characterized by cornified epithelial cells; metestrus was characterized by both cornified epithelial cells and leukocytes; and diestrus was characterized by a predominance of leukocytes and nucleated epithelial cells.

### Fertility Assessment

To examine fertility, female mice including DHEA treated LKO, WT, LTg and LCtrl mice were paired with proven C57BL/6J stud males for mating. After confirming vaginal plug, the few female mice were sacrificed to confirm pregnancy as previously described.^[^ ^45^ ^]^

### Glucose Tolerance Test and Insulin Tolerance Test

For glucose tolerance test (GTT), mice were fasted for 12 h. After measurement of the level of fasting glucose through tail vein, mice were intraperitoneally injected D‐glucose (2 g kg^−1^ body weight), the blood glucose was tested at 15/30/60/90/120 min respectively. For insulin tolerance tests (ITT), after 6 h of fasting, the mice were intraperitoneally injected with insulin (1 IU kg^−1^) and the blood glucose were tested at 15/30/60/90/120 min respectively.

### Histology and Follicles Counting

Tissues were fixed in 4% paraformaldehyde in PBS for at least 24 h. Then, tissues were dehydrated, embedded in paraffin, and sectioned at 5 µm and subjected to H&E staining. For estimation of ovarian follicles, five serial H&E staining sections were used to quantify follicular numbers as previously described.^[^ ^54^ ^]^

### ELISA Assay

Murine whole blood was collected and centrifuged at 3000 r.p.m. for 15 min at 4 °C. Then, the serum of each sample was aliquoted and kept at −80 °C. The concentration of mouse serum LH (JL10432‐96T, Shanghai Jiang Lai Bioengineering Institute), FSH (JL10239‐96T, Jiang Lai Bioengineering Institute), testosterone (JL12550, Jiang Lai Bioengineering Institute) and DHT (JL10916, Jiang Lai Bioengineering Institute) and the concentration of medium supernatant of testosterone (JL10895, Jiang Lai Bioengineering Institute) and DHT (JL12546, Jiang Lai Bioengineering Institute) were determined by ELISA following manufacturer's instructions.

### Isolation of Primary Hepatocytes

Murine primary hepatocytes were isolated via two‐step liver perfusion method.^[^ ^55^, ^56^ ^]^ Briefly, mice were anesthetized with intraperitoneal injection of pentobarbital sodium (10 mg kg^−1^ body weight), the liver was perfused by Perfusion medium (17701‐038; GIBCO) and then by Liver Digest Medium (17703‐034; GIBCO). The livers were extirpated and prepared for cell suspension. For purification, liver cell suspension was filtered through 100 µm cell strainers and then resuspended in Percoll/ RPMI 1640/10 ×  PBS (1:1:0.1) mixture and centrifuged at 300 × g for 10 min at 4 °C. Cells were washed 3 times with ice‐cold RPMI 1640 and centrifuged at 50 g for 2 min at 4 °C. After assessment of cell viability by the Trypan blue, cells were counted and harvested for further treatment.

### Cell Culture and Treatment

Murine primary hepatocytes were cultured in RPMI 1640 medium (H10394; Invitrogen) with 10% FBS (10 099 158; GIBCO) on rat tail collagen‐coated 6 well plates. For DHEA treatment, cells were treated with 20 µM DHEA for 48 h and harvested for further processing. hiHeps were cultured in DMEM/F12 (11 320 033; GIBCO) with 10 µM Dex (D4902; Sigma–Aldrich), 40 ng mL^−1^ recombinant human TGFα (100‐16A, Peprotech), 40 ng mL^−1^ recombinant human EGF (AF‐100‐15, Peprotech) on 6 cm dish. The cells were treated with 20 µM DHEA for 24 h and harvested for further processing.

For knockdown of *Klf9*, cells were transfected with indicated adenoviruses (1.0 × 10^7^ infection particles per cell of a 6‐well plate) for 24 h. ^(^ ^25^ ^)^ After infection for 24 h, cells were replaced with fresh medium for further treatment.

### Western Blotting

Protein samples from tissues or cells were lysed in RIPA buffer (20 mM Tris‐Cl pH 7.5, 140 mM NaCl, 1 mM CaCl_2_ and MgCl_2_, 10 mM NaF, 1% NP‐40, 10% glycerol, 2 mM Na‐Vanadate, and 1 mM PMSF) with protease inhibitor cocktail (P8340, Sigma–Aldrich). The concentration of total protein was determined by BCA Kit (23 225, Thermo Scientific). Equal amounts of denatured proteins were separated by SDS‐PAGE and transferred to PVDF membranes. After blocking with skim milk in TBST, the membranes were incubated with primary antibody: Anti‐KLF9 (1:1000; A7196; Abclonal), Anti‐FLAG (1:1000; F1804; Sigma–Aldrich), Anti‐β‐Tubulin (1:4000; KM9003T; Tianjin Sungene Biotech), Anti‐mouse SRD5A1(1:1000; A14787; Abclonal), Anti‐human SRD5A1(1:1000; 26001‐1‐AP; Proteintech), HSD3B2(1:1000; A1823; Abclonal) at 4 °C overnight. Then, the membranes were incubated with HRP‐conjugated second antibodies: HRP Goat Anti‐Rabbit IgG (1:5000; AS014; Abclonal), HRP Goat Anti‐Mouse IgG (1:5000; AS003; Abclonal). The bands were quantified by Image J with normalization to Tubulin.

### Quantitative Real‐Time PCR

Total RNAs of cells or tissue were extracted by TRIzol reagents (15 596 026, Invitrogen). RNA quantity and purity were determined by NanoDrop. After synthesis of cDNA using HiScript IV 1st Strand cDNA Synthesis Kit (R412‐01, Vazyme), qPCR was performed using ChamQ Universal SYBR qPCR Master Mix (Q711‐02, Vazyme) with specific primers according to the manufacturer's instructions. Then, the relative mRNA level of target gene expression was calculated by comparative CT method((2^−ΔΔCt^). *36B4* or *β‐actin* were used as housekeeping gene. Primer sequences used were listed in Table S1 (Supporting Information).

### Bulk RNA‐Seq

RNA from tissues were extracted using TRIzol reagents (15 596 026, Invitrogen) and treated with DNaseI (EN0523, Thermo Fisher). cDNA library was generated according to the manufacturer's protocol (FC‐131‐1024, Illumina). Samples were sequenced with a DNBSEQ system. Raw reads were filtered and clean reads were aligned with reference genome Mus musculus (GRCm38) using bowtie2.

### CUT&Tag

CUT&Tag assay was performed according to the Hyperactive Universal CUT&Tag Assay Kit for Illumina (Vazyme, #TD903‐01). Briefly, a total of 30 000 primary hepatocytes per sample were harvested and washed twice by PBS. After centrifugation, the washed cells were incubated with active conA beads in wash buffer for 10 min at room temperature. Then, each sample was incubated with primary antibody [Anti‐ FLAG (F1804, Sigma–Aldrich), Anti‐Androgen Receptor (ab108341, abcam), Anti‐Rabbit IgG (ab172730, abcam), Anti‐Mouse IgG (5415, CST)] at a 1:50 dilution at 4 °C overnight. After incubation with secondary antibody [Donkey anti‐Rabbit IgG (Ab206‐01‐AC, Vazyme), Goat anti‐mouse IgG (Ab208‐01‐AA, Vazyme)] at a dilution of 1:100 followed by incubation with pA/G‐Tnp for 1 h at room temperature on a rotator, each sample was added with TTBL in Dig‐300 Buffer incubated at 37 °C 1 h for fragmentation. Next, the DNA was extracted by incubation with a mixture of proteinase K, buffer L/B and DNA extract beads at 55 °C for 10 min followed by washing with buffer WA and WB. For library amplification, a volume of 25 µl of 2 × CAM with 15 µl purified DNA were added and mixed. The PCR amplification condition as following: 72 °C for 3 min; 95 °C for 3 min; 98 °C for 10 s; 60 °C for 5 s; 15 cycles of 98 °C for 10 s; 60 °C for 5 s; final extension at 72 °C for 1 min; and hold at 4 °C. Then, the products were cleaned up with VAHTS DNA Clean Beads and 80% ethanol. The DNA libraries were quantified and sequenced on the Illumina HiSeq 2500 platform.

### ChIP‐qPCR

ChIP‐qPCR was carried out following the manufacture's protocols (SimpleChIP Plus Sonication Chromatin IP Kit, 56383, CST) with minor modifications. Briefly, hepatocytes were washed with PBS three times and fixed with 1% formaldehyde at room temperature for 15 min. Then, the cross‐linking was quenched by glycine. Cells were subjected to sonication and then the sheared chromatin was immunoprecipitated with primary antibody [Anti‐ FLAG (F1804, Sigma–Aldrich), Anti‐Androgen Receptor (ab108341, abcam), Anti‐Rabbit IgG (ab172730, abcam), Anti‐Mouse IgG (5415, CST)]. After reversal of Cross‐links, the eluted and purified DNA was served as template to process to real‐time PCR. The primer sequences can be found in the Table S2 (Supporting Information).

### Statistical Analysis

Data were presented as mean ±SD. Statistical analysis were assessed by two‐tailed unpaired Student's test for two groups and Two‐way ANOVA for multiple groups in this study. Statistics were calculated using Graphpad Prism version 8. *P < 0.05* was considered to be statistically significant (*, *p≤0.05*; **, *p≤0.01*, *** *p≤0.001* and **** *p ≤ 0.0001*).

## Conflict of Interest

The authors declare no conflict of interest.

## Author Contributions

J.S., Z.G. and G.J. contributed equally to this work. H.F., (Yaolin Zhang) Y.Z., Y.C. and H.R. conceived the study and designed the project; J.S., (Yaolin Zhang) Y.Z., Z.G., G.J, X.Y., and (Yujie Zhang) Y.Z. performed the experiments. J.W., Y.Z. (Yinliang Zhang) performed data analysis. C.H. helped with manuscript revision. (Yaolin Zhang) Y.Z. and H.F. wrote the manuscript. All authors reviewed and contributed to the manuscript. The order among co‐first authors was assigned based on contributions.

## Supporting information



Supporting Information

## Data Availability

The data that support the findings of this study are available from the corresponding author upon reasonable request.
