# Supplementary material for: Hepatic KLF9 Deficiency Inhibits Dehydroepiandrosterone (DHEA)‐Induced Polycystic Ovary Syndrome via Liver‐Ovary Axis
Source: Adv Sci (Weinh). 2025 Sep 23;12(48):e08240. doi: 10.1002/advs.202508240 (PMC12752638; doi:10.1002/advs.202508240)
Supplement: Supplementary file 1 — Supporting Information [file ADVS-12-e08240-s001.docx]

**Supplemental Data**

**
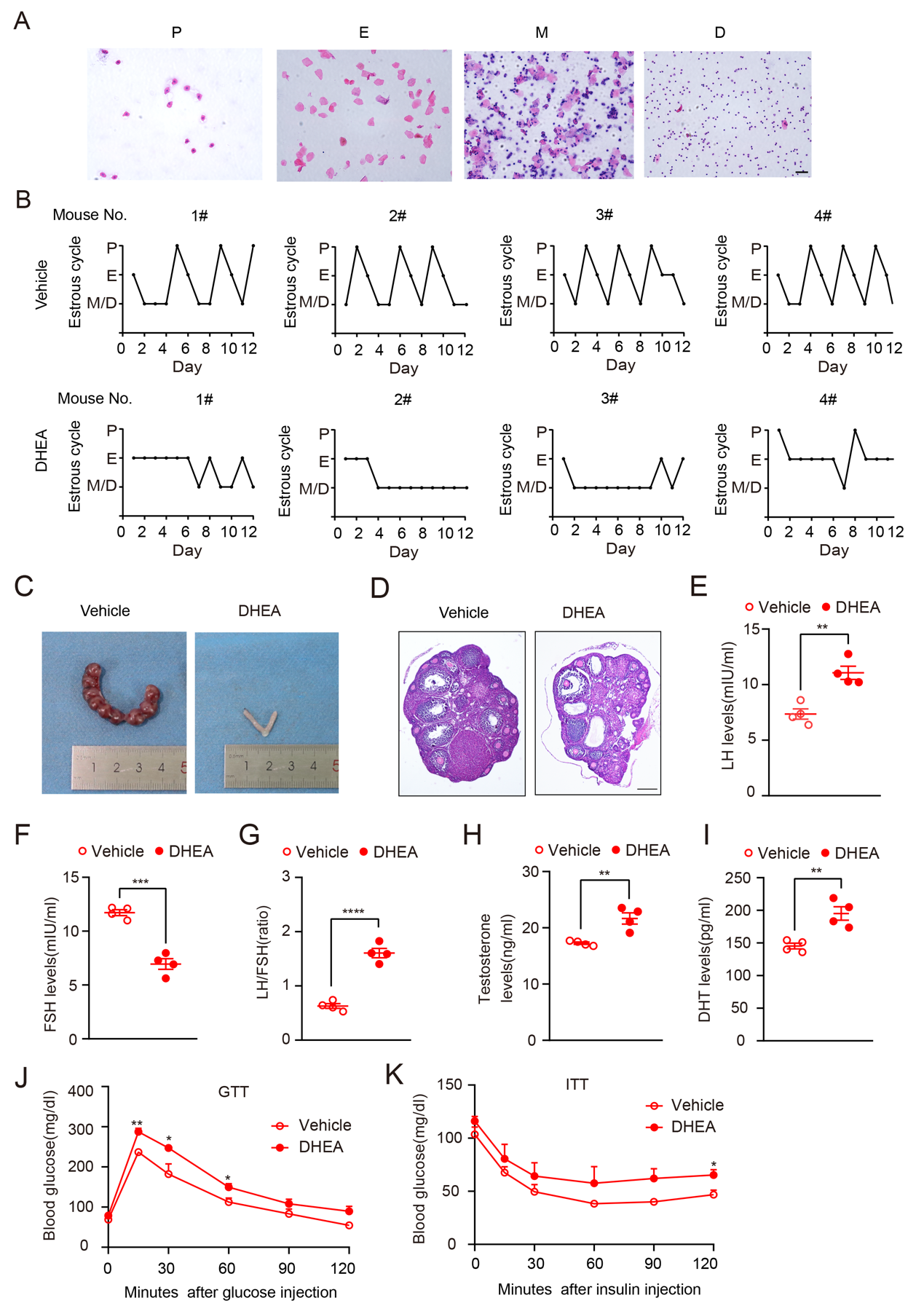
**

**Supplemental Figure S1**. **Establishment of a mouse model of PCOS by DHEA.**

(A) Representative morphology of estrous cyclicity in female C57BL/6J mice by H&E staining. P, proestrus; E, estrus; M, metestrus; D, diestrus. Scale bar: 50 μm. (B) Representative estrous cyclicity of mouse treated by vehicle and DHEA (n=4 per group). P, proestrus; E, estrus; M/D, metestrus/diestrus. (C) Representative images of fertility test. (D) Representative morphology of ovary stained by hematoxylin and eosin. Scale bar: 200 μm. (E-G) ELISA-based quantification of LH, FSH the and the ratio of LH/FSH (n=4 per group) in the serum of mice administrated with DHEA and vehicles, respectively. (H-I) ELISA-based quantification of testosterone and DHT in the serum of mice administrated with DHEA or vehicles. (J-K) Glucose tolerance test and insulin tolerance test results (n=4 per group). Data are presented as mean ±SD. Statistical analysis was performed using two-tailed unpaired *t*-tests. *p < 0.05, **p < 0.01, ***p < 0.001 and ****p < 0.0001.


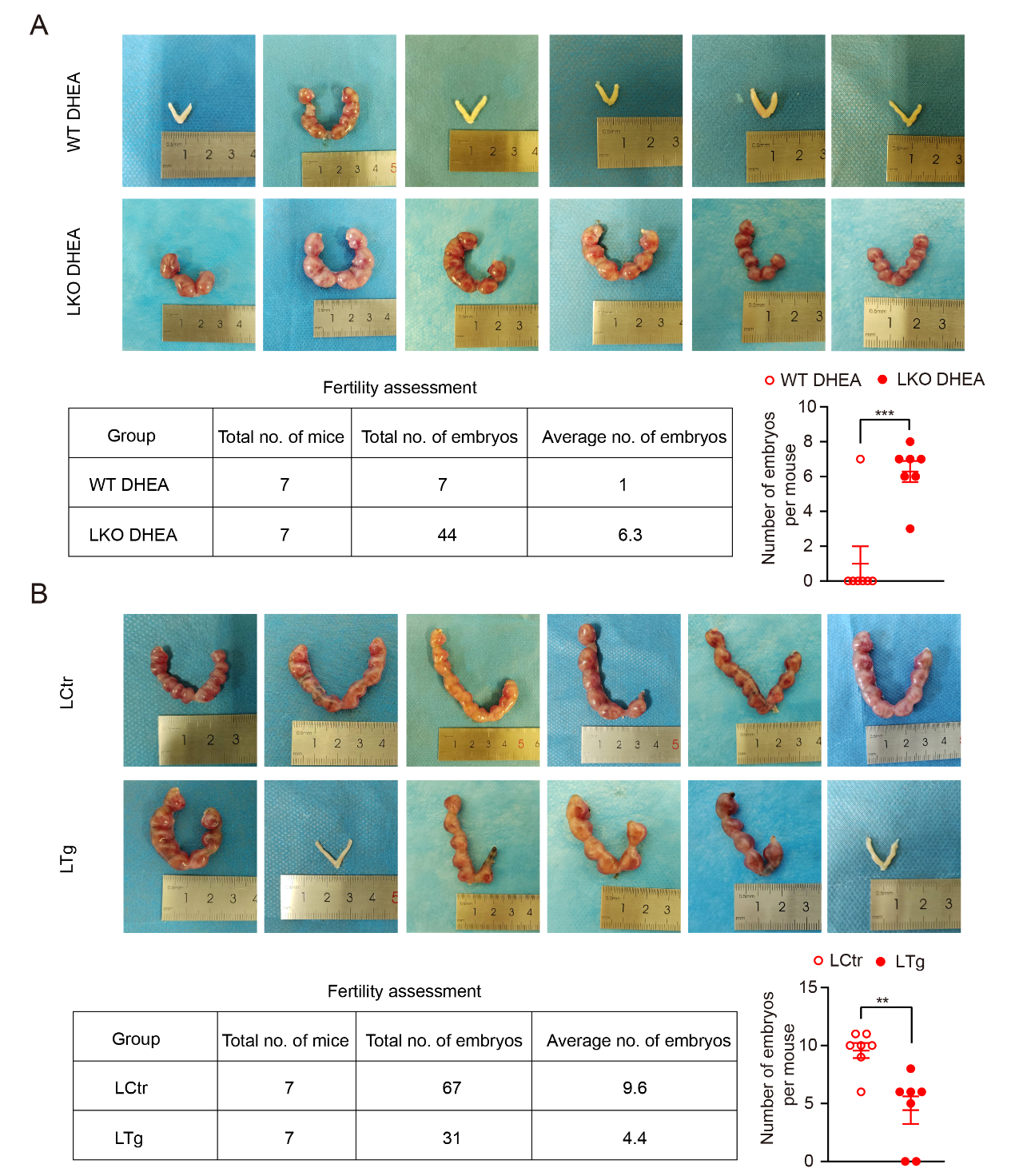


**Supplemental Figure S2. Fertility assessment of LKO (treated with DHEA) and** **LTg mice.**

(A). Images showing that the embryos of LKO and WT mice in the setting of PCOS-induced by DHEA. The table showing the quantitative analysis of embryos from WT and LKO mice treated with DHEA. (B). Images showing that the embryos of LCtr and LTg mice. The table showing the quantitative analysis of embryos from LCtr and LTg mice.


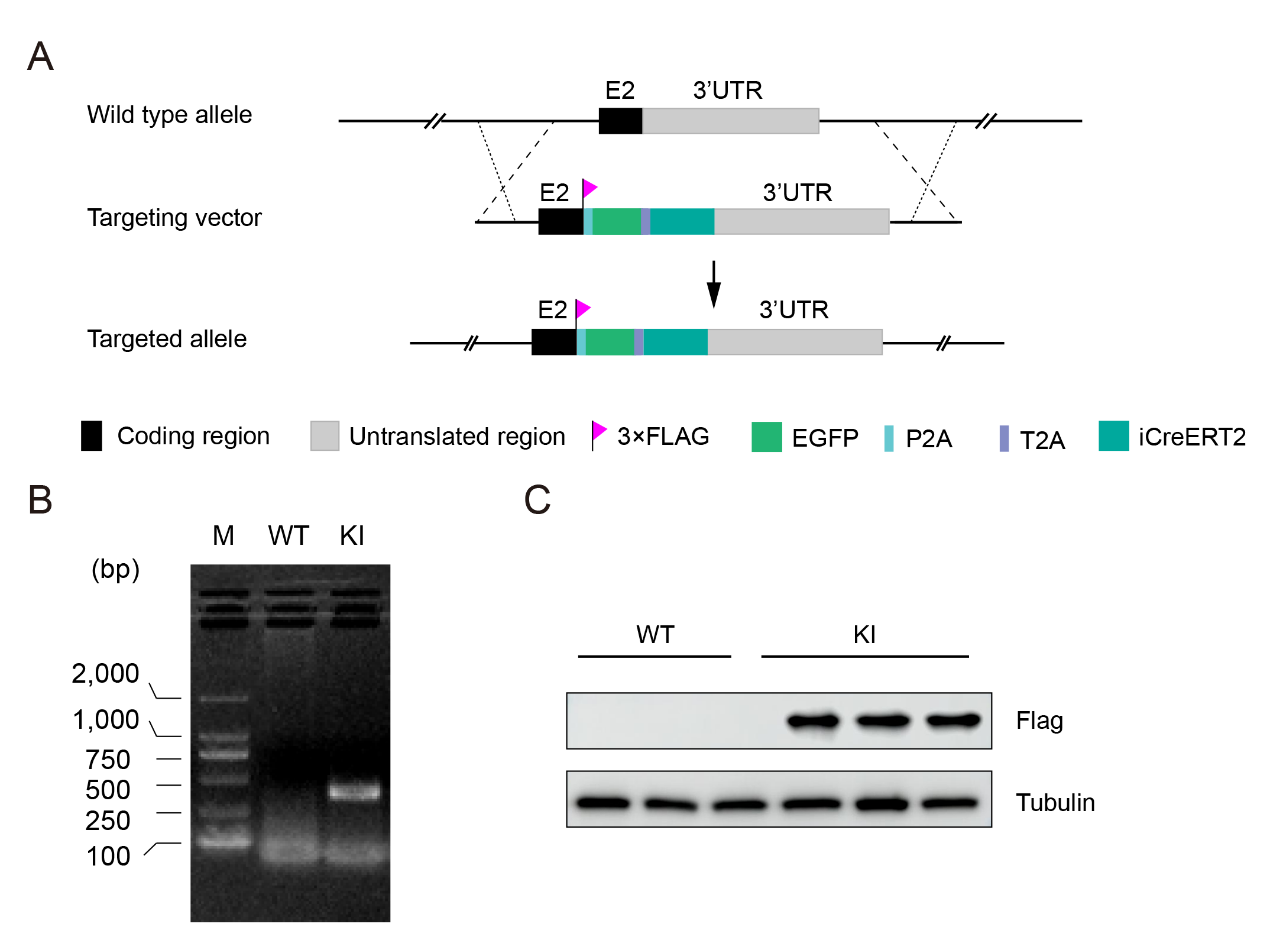


**Supplemental Figure S3. Generation of 3×FLAG-KLF9 constitutive knock-in mice.**

(A) Schematic illustration of the 3×FLAG-KLF9 constitutive knock-in mice. (B) PCR genotyping of genomic DNA confirmed germline transmission of the FLAG-tag. (C) Immunoblot analysis of FLAG expression in the liver of 3×FLAG-KLF9 constitutive knock-in mice and control mice.

**Table1. Primer sequences**

| Gene | Species | Primer sequence |
| --- | --- | --- |
| *36B4* | mouse | forward 5’-GAGGAATCAGATGAGGATATGGGA-3’ |
|  |  | reverse 5’- AAGCAGGCTGACTTGGTTGC-3’ |
| *Klf9* | mouse | forward 5’- CGAGCGGCTGCGACTACCTG -3’ |
|  |  | reverse 5’- GGGCTGTGGGAAGGACTCGAC -3’ |
| *Srd5a1* | mouse | forward 5’- GAGTTGGATGAGTTGCGCCTA -3’ |
|  |  | reverse 5’- GGACCACTGCGAGGAGTAG -3’ |
| *Hsd3b2* | mouse | forward 5’- GGTTTTTGGGGCAGAGGATCA -3’ |
|  |  | reverse 5’- GGTACTGGGTGTCAAGAATGTCT -3’ |
| *Hsd3b3* | mouse | forward 5’- TCAAACCTGAAACCAGGGAG -3’ |
|  |  | reverse 5’- TCAGACCATGTGCTTTCACG -3’ |
| *Hsd3b1* | human | forward 5’- CACATGGCCCGCTCCATAC -3’ |
|  |  | reverse 5’- GTGCCGCCGTTTTTCAGATTC -3’ |
| *Hsd3b2* | human | forward 5’- AGAACGGCCACGAAGAAGAG -3’ |
|  |  | reverse 5’- TGGGTCTTAACGCACAAGTGT -3’ |
| *Srd5a1* | human | forward 5’- TCTGATGCGAGGAGGAAAGC -3’ |
|  |  | reverse 5’- CATGCCCGTTAACCACAAGC -3’ |
| *Klf9* | human | forward 5’- GCCGCCTACATGGACTTCG -3’ |
|  |  | reverse 5’- GGATGGGTCGGTACTTGTTCA -3’ |
| *β-actin* | human | forward 5’- CATGTACGTTGCTATCCAGGC -3’ |
|  |  | reverse 5’- CTCCTTAATGTCACGCACGAT -3’ |
|  |  |  |

**Supplemental Table2. Primer sequences for ChIP-qPCR.**

| Target genes | Species | Primer sequence |
| --- | --- | --- |
| *Klf9-1* | mouse | forward 5’- CTCTGGCTGGGGATGCATAG -3’ |
|  |  | reverse 5’- CTGTGCGAGTGGAGATGACT -3’ |
| *Klf9-2* | mouse | forward 5’- GGGGTAGGGTAGTCAAATGGC -3’ |
|  |  | reverse 5’- TGAAAAGTAGCCGCCATCCA -3’ |
| *Srd5a1* | mouse | forward 5’- AGCGAGAGTCTGTTTCGAGC -3’ |
|  |  | reverse 5’- TTGGCTGAGAGCTCCTTTGG -3’ |
| *Hsd3b3* | mouse | forward 5’- AACACCCACATAGGCTCACA -3’ |
|  |  | reverse 5’- CCAAGAATGCCACACCTCCT -3’ |
|  |  |  |
